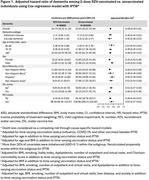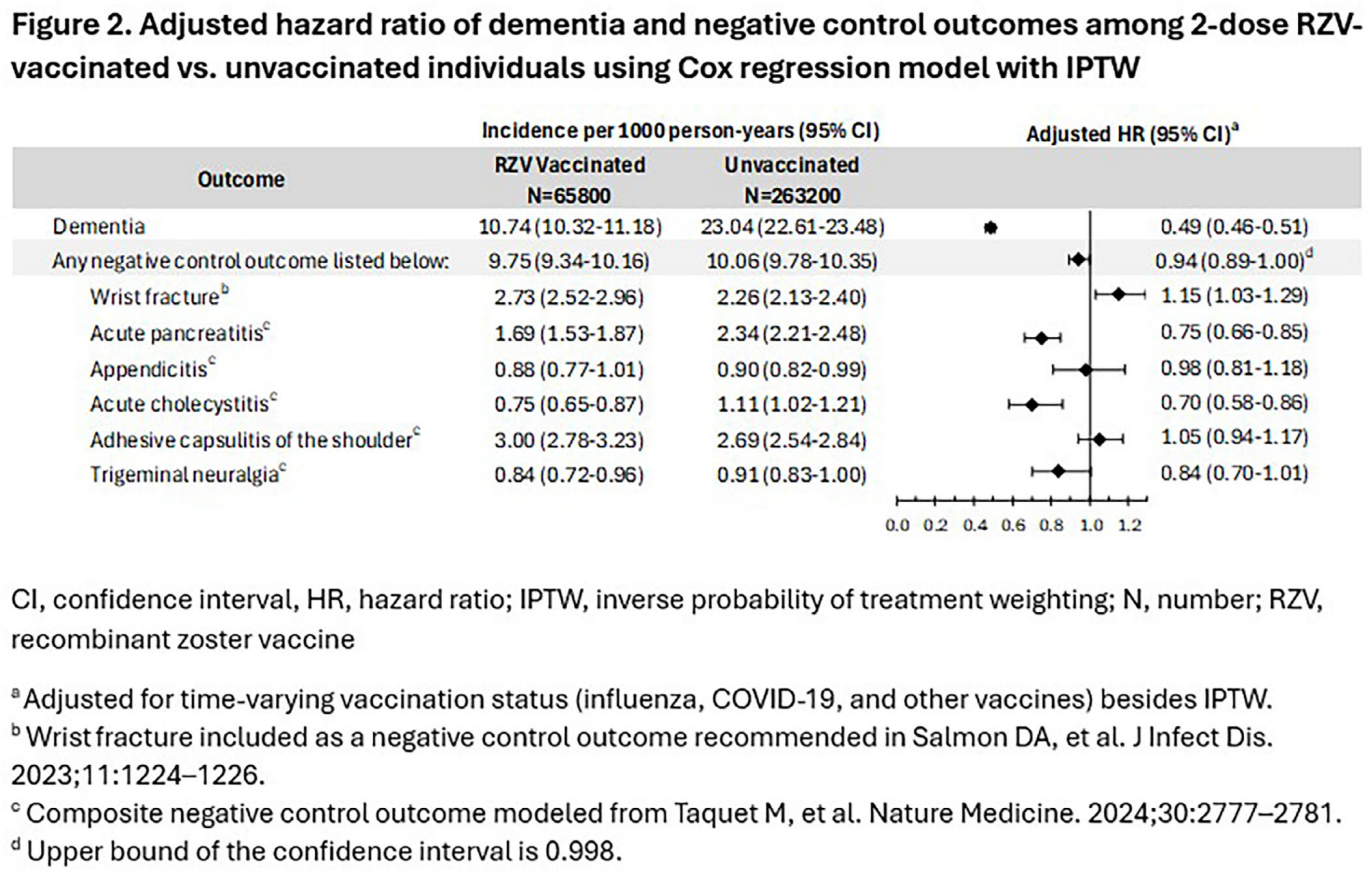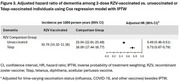# Recombinant zoster vaccine is associated with lower risk of dementia in adults aged ≥65 years

**DOI:** 10.1002/alz70861_108095

**Published:** 2025-12-23

**Authors:** Emily Rayens, Lina Sy, Lei Qian, Bradley Ackerson, Julia Tubert, Yi Luo, Punam Modha, Raul Calderon, Elizabeth Chmielewski‐Yee, Driss Oraichi, Huifeng Yun, Carol Koro, Hung Fu Tseng

**Affiliations:** ^1^ Kaiser Permanente Southern California, Pasadena, CA USA; ^2^ GSK, Rockville, MD USA; ^3^ GSK, Collegeville, PA USA

## Abstract

**Background:**

Vaccines to prevent herpes zoster, historically zoster vaccine live (ZVL), have been associated with reduced dementia risk. We evaluated the association between recombinant zoster vaccine (RZV) and reduction in all‐cause dementia in adults aged ≥65 years at Kaiser Permanente Southern California.

**Methods:**

We conducted a retrospective matched cohort study of adults aged ≥65 years who received 2 doses of RZV 4 weeks‐6 months apart between 1 April 2018 and 31 December 2020 and were matched 1:4 with unvaccinated individuals on age group, sex, race/ethnicity, and history of ZVL, with no dementia diagnoses or dementia medications prior to or within 6 months of index date (second dose date among vaccinated; same date for matched unvaccinated). Follow‐up started 6 months after index date until dementia identification by ≥1 ICD‐10 code in electronic health records, receipt of zoster vaccine, membership termination, death, or end of follow‐up (31 December 2023), whichever came first. Cox regression with inverse probability of treatment weighting was used to estimate adjusted hazard ratios (aHRs). To evaluate residual confounding, we assessed the relationship between RZV and a composite negative control outcome (NCO). To evaluate potential healthy vaccinee bias, we compared dementia incidence in RZV‐vaccinated individuals versus Tdap‐vaccinated individuals.

**Results:**

This study included 65,800 individuals vaccinated with 2 RZV doses and 263,200 unvaccinated (57.6% female, 61.2% non‐Hispanic white, mean age 73.3 years). The mean follow‐up time in vaccinated and unvaccinated individuals was 3.4 and 1.8 years, respectively. The incidence rate of dementia among vaccinated was 10.74 (95% confidence interval [CI]: 10.32‐11.18) per 1000 person‐years compared to 23.04 (95% CI: 22.61‐23.48) among unvaccinated individuals. The aHR of 2 doses of RZV against dementia was 0.49 (95% CI: 0.46‐0.51; Figure 1); aHRs were comparable across age and racial/ethnic groups. The aHR for the NCO was 0.94 (0.89‐1.00; Figure 2). The aHR of 2 doses of RZV compared to Tdap was 0.73 (0.67‐0.79; Figure 3).

**Conclusions:**

2 RZV doses were significantly associated with a reduction in risk of dementia in adults aged ≥65 years. After accounting for healthy vaccinee bias, RZV vaccination remained associated with a statistically significant lower risk of dementia.